# A group analysis using the Multiregression Dynamic Models for fMRI networked time series

**DOI:** 10.1016/j.jspi.2018.03.004

**Published:** 2019-01

**Authors:** Lilia Costa, James Q. Smith, Thomas Nichols

**Affiliations:** a Universidade Federal da Bahia, Brazil; b The University of Warwick, UK; c Oxford Big Data Institute, Li Ka Shing Centre for Health Information and Discovery, Nuffield Department of Population Health, University of Oxford, UK

**Keywords:** Multiregression Dynamic Model, Bayesian network, Group analysis, Cluster analysis, Functional Magnetic Resonance Imaging (fMRI)

## Abstract

Connectivity studies of the brain are usually based on functional Magnetic Resonance Imaging (fMRI) experiments involving many subjects. These studies need to take into account not only the interaction between areas of a single brain but also the differences amongst those subjects. In this paper we develop a methodology called the *group-structure* (GS) approach that models possible heterogeneity between subjects and searches for distinct homogeneous sub-groups according to some measure that reflects the connectivity maps. We suggest a GS method that uses a novel distance based on a model selection measure, the Bayes factor. We then develop a new class of Multiregression Dynamic Models to estimate individual networks whilst acknowledging a GS type dependence structure across subjects. We compare the efficacy of this methodology to three other methods, virtual-typical-subject (VTS), individual-structure (IS) and common-structure (CS), used to infer a group network using both synthetic and real fMRI data. We find that the GS approach provides results that are both more consistent with the data and more flexible in their interpretative power than its competitors. In addition, we present two methods, the Individual Estimation of Multiple Networks (IEMN) and the Marginal Estimation of Multiple Networks (MEMN), generated from the GS approach and used to estimate all types of networks informed by an experiment —individual, homogeneous subgroups and group networks. These methods are then compared both from a theoretical perspective and in practice using real fMRI data.

## Highlights

• The group-structure (GS) approach models possible heterogeneity between subjects.
• The search for distinct homogeneous groups is based on the connectivity maps.
• All four methods for group network were compared using synthetic and real fMRI data
• IEMN and MEMN estimate individual, homogeneous subgroups and group networks.

## 1 Introduction

Functional MRI measures changes in metabolism that occur in brain tissue. Using fMRI, it is possible to locate the areas of the brain which are responsible, for example, for memory, language and hearing. This technique can now provide an image of the brain which has both good spatial and temporal resolution. Furthermore measurements can now be obtained both safely and noninvasively (Poldrack et al., 2011). Although fMRI experiments are widely used to understand how the brain works in response to certain tasks, they also provide a very useful tool to help understand the working of the brain when a person is resting (Raichle, 2010). These mechanisms are important to understand because they provide a backcloth around which to measure the impact of activity a brain function. These resting-state experiments are conducted by having the subject remain in a state of quiet repose and will be the focus of this paper.

One way to understand the brain during this resting-state is to study the connectivity among different cerebral areas, *i.e.*, to assess how one neural system influences another Smith et al. (2011), Friston (2011), Poldrack et al. (2011). The interpretation of this connectivity depends strongly on the statistical procedure used to estimate it. For instance, *functional connectivity* is defined as the correlation among measurements of neuronal activity of different areas (Friston, 2011). Although there is a correlation between the two regions, this does not imply that one region directly influences the other one. Therefore an *effective connectivity* is defined based on hypotheses concerning potential causal relationship in the brain (*e.g.* Spirtes et al., 2000; Pearl, 2000). Mechelli et al. (2002) clarifies the difference between functional and effective connectivity by stating that functional connectivity is defined as the temporal correlations among neurophysiological events in different neural systems, whereas effective connectivity is defined as the influence that one neural system exerts over another.

**Fig. 1:**
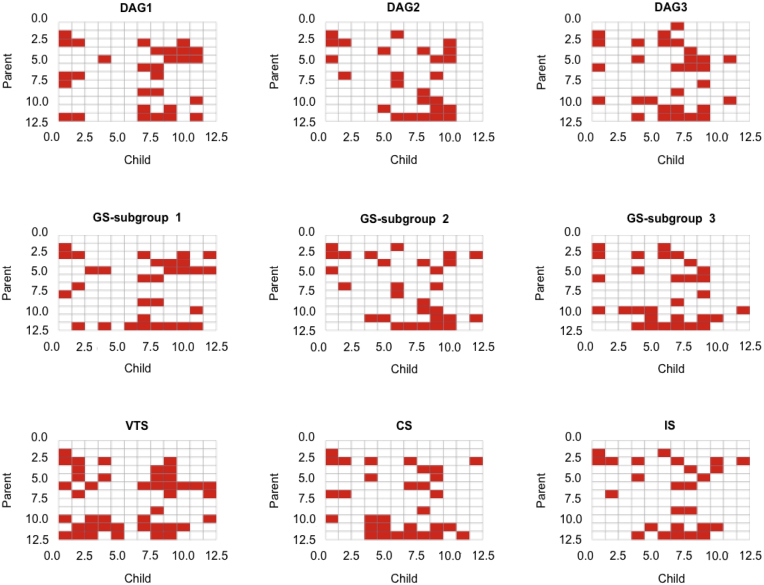
Data was simulated considering these three difference graphical structures: DAG1, DAG2 and DAG3 (in the first row). The GS approach found three groups and estimated graphs are in the second row. The estimated DAGs for the VTS, CS and IS approaches are in the third row.

Connectivities are usually studied using different classes of graphical model (see *e.g.* Sporns, 2011). Such a graph consists of nodes and edges in which the latter represents connectivity between pairs of nodes sited at a voxel or defined brain region. Once such nodes have been defined, an integration study, *e.g.* Bayesian network (BN; Korb and Nicholson, 2004), can be used to find edges. These edges generally are either undirected when the model simply reflects some dependence of measurements at two connected sites, or directed where the receiving node (the child) is hypothesised to be influenced by the donating node (the parent). *Directed acyclic graphs* (DAGs) are graphs whose edges are all directed and no path starts and ends at the same node, see the examples of DAG in Fig. 1. The focus of this paper will be on studying effective connectivity through a particular class of DAG models.

One useful family of statistical models that has recently been used to study brain connectivity is the Multiregression Dynamic Model (MDM; Costa et al., 2015). In this model the connections are represented by parameters that vary over time. As a result, the MDM generalises the class of BNs into a useful class where connectivity strengths between two nodes are hypothesised to change in the time. In contrast to most methods that use Granger causality to estimate effective connectivity, in this model the edges denote direct contemporaneous relationships that might exist between nodes.

Despite its flexibility, the MDM can be supported by a conjugate analysis and its predictive likelihood has a closed form which allows us to perform fast model selection. In Costa et al. (2015) we provide an efficient search over this large class of networks based on an integer programming algorithm. The so-called Multiregression Dynamic Model-Integer Programming Algorithm (MDM-IPA) performed well in detecting the presence of a network connection and also in distinguishing the directionality of the relationships between the brain regions. Here, we also search over another large class of models, the Multiregression Dynamic Model-Directed Graph Model (MDM-DGM) class, which does not demand the acyclic constraints required for the vanilla MDM and searches the larger class of directed graphs. This appears to perform even better in applications in neuroscience than the vanilla MDM. This is because unlike the MDM-IPA it is able to model a bidirectional communication between brain regions which typically exists in this domain. The application of these search methods into real resting-state and task fMRI data can be seen *e.g.* in Costa (2014), Costa et al. (2015), Harbord et al. (2016) and Costa et al. (2017).

However connectivity studies are usually based on fMRI experiments using many subjects and not just one. Therefore, ideally any analyses should take into account not only the interaction between the areas of one single brain but also the differences among subjects. In this way, in multi-subjects experiments, generally there are three main analyses of interest: (i) to estimate individual networks; (ii) to compare them and then verify if the group of people are homogeneous. If this is not the case then we can add into the model explanatory variables such as gender, age and diseases to explain the heterogeneity we discover; (iii) to estimate a network that represents homogeneous subgroups and the entire group of these individuals.

The purpose of this paper is to present some new approaches that address these three issues described above. For simplicity, we have organisedthis paper into two parts. In the first part, corresponding to Sections from 2 to 5, we compare four methods used to estimate group networks. We also demonstrate the importance of doing this and explicitly how to study the homogeneity of a group. In standard fMRI studies different subjects can display very different relational maps of connectivity from each other. Therefore whilst combining subjects can be a very helpful way to extract information about the whole group, any assumption that implies subjects a posteriori exchangeable is very contentious in this domain. Fortunately the algorithm we use to search for the best fitting models for a single subject can also be used as a basis for a separation measure for first clustering subjects into those exhibiting similar connectivity relationships between the regions of their brain. We demonstrate here that by using this clustering method (to first determine promising subgroups we want to combine) performs much better than any current direct combination method.

The use of group analysis to assess the integration of activity in brain regions – as we perform here – has two advantages (Mechelli et al., 2002). First, it is possible to investigate directly which connections are different across subjects. For instance, some patients may not have a particular connection that exists in healthy people. Alternatively, the connection strength may vary according to age. Second, by allowing the measurement from each individual subject to be modelled, the degrees of freedom that exist over for example typical subject analyses can potentially improve the statistical power of any analysis we propose.

Most group analysis methods do not first verify whether or not the group of subjects is a sample of populations with different connectivity patterns. Instead, the standard *group-structure* approaches (GS) aim to find homogeneous sub-groups according to connectivity maps. Here we suggest an analysis of this type which allows a novel distance based on the model selection measure, the Bayes factor (Jeffreys, 1961). The first step to analyse this group of subject is to calculate and record the dynamic regression scores for all individuals so as to find MAP models between our classes. Of course, this is a huge task. However, once we have done this we can then use the scores we calculate to determine various types of aggregate graphs relatively cheaply. In this paper we show how within our dynamic framework such aggregate graphical models can be discovered which depict useful relationships both within and between the brain images of different subjects.

We note that although the GS approach developed here is applied to analyses using the class of MDM models, it can be used with any other probabilistic graphical model. We show here that, using both synthetic and real fMRI data, the GS approach provides results that are more scientifically plausible and that are more sensitive to potential heterogeneities within a population than virtual-typical-subject (VTS), individual-structure (IS) and common-structure (CS) approaches.

Therefore, in this first part of the paper, we discussed methods used to infer only the group network. Then, in the second part of this work, corresponding to Section 6, we extend this discussion to all types of networks informed by an experiment — individual, homogeneous subgroups and group networks. We define the Individual Estimation of Multiple Networks (IEMN) approach which is basically the GS approach showed in the first part, *i.e.* the individual networks are estimated independently whilst the subgroup networks made up of homogeneous subjects are estimated as a function of the same graphical structure for all individuals. We then compare this to the new approach called the Marginal Estimation of Multiple Networks (MEMN) that enables us to infer individual networks considering the information of other subjects. This method searches both individual and subgroup networks considering a distance between homogeneous subjects, as shown below.

In theory, MEMN provides more precise results than IEMN, because when the graphical structure is unknown, the learning algorithm adds more uncertainty to the inferential process. In this sense the use of information from other subjects in the learning individual networks, as the MEMN does, can therefore improve this estimation process. We note that Oates et al. (2014a), Oates et al. (2014b) presented a similar method to MEMN. These all the individual and subgroup networks are estimated simultaneously, and then the denser graphs are penalised. However network learning is more complex and rather less transparent compared to the one we discuss here. IEMN and MEMN are compared in theory and in practice using real fMRI data. Short description and some references for all methods used in this work can be seen in Appendix A.

This paper is structured as follows. Section 2 provides a brief literature review of some methods used for group analysis. Section 3 describes these group analysis methods in the context of the MDM. Section 4 compares these four methods using synthetic data whilst Section 5 analyzes the group of subjects considering real resting-state fMRI data. Section 6 presents methods to estimate both individual and group networks, IEMN and MEMN, and a comparison between them, and finally conclusions are given in Section 7.

## 2 A short review of group analysis methods

Many fMRI experiments with multiple subjects have been conducted recently. In general, four approaches which deal with multi-datasets may be found in the neuroimaging literature (*e.g.* Mechelli et al., 2002; Li et al., 2008; Ramsey et al., 2010; Gates and Molenaar, 2012). The first approach is the *virtual-typical-subject* (VTS). This approach ignores inter-subject variability, assuming that the information from different datasets come from the same subject. This “typical subject” can be found by calculating the average of observed variables for every node over subjects or concatenating the datasets, so that methods designed for a single individual can be used Zheng and Rajapakse (2006), Rajapakse and Zhou (2007), Li et al. (2008). Of course, when datasets are concatenated, the number of data points per node increases. Consequently the degree of freedom for estimating the parameters is higher. However, the assumptions underpinning this approach, *i.e.* “variations in connectivity from subject to subject are random, well-behaved and uninteresting”, are not always true (Mechelli et al., 2002). Moreover, the variability of *concatenated data* may be significantly higher than individual variability whilst the variability of *averaged data* may be very much lower than the usual variability found for each subject. Therefore, the results of this group-based analysis may not reflect some of the features found in the individual context (Gonçalves et al., 2001). In addition, it is not possible to compare the interactions according to different characteristics, such as task performance or gender.

Gates and Molenaar (2012) give two reasons why VTS is not suitable for modelling a brain network. The first concerns the connectivity strength. It is generally accepted that this is expected to vary between subjects. However, some researchers want to study the relationship between this *connectivity strength* and *disease level* and this cannot be addressed using this method. Second, the communication pattern among brain regions may well differ from individual to individual. For instance, in a study of fMRI activation pattern related to writing, three of five regions showed inconsistent results across subjects (Sugihara et al., 2006). To address these problems a second method, *common-structure* (CS), and a third method, *individual-structure* (IS), have been proposed.

The CS approach considers the same network structure but allows the parameters to differ between subjects. The connectivity strengths are expected to vary over subjects due to measurement error or the individual characteristic of influences from one region to another. An example of this approach is the *Independent Multiple-sample Greedy Equivalence Search* (IMaGES) which uses BIC scores to find a Markov equivalence class, basically summing the scores over subjects who are assumed to share the same graphical structure (Ramsey et al., 2010). Clearly the CS approach cannot, therefore, allow for the possibility that the pattern of connectivity may diverge over subjects. However, such a divergence may plausibly happen. For example, in a resting-state experiment when people are free to think of anything, someone may use their memory whilst others may do mental arithmetic. Another reason why the assumptions on which such methods are based might be violated is that a group of patients may well have a disease with different degrees of severity. This could then result in different connections as well as different connectivity strengths arising between brain regions (Li et al., 2008).

The next approach, *individual-structure* (IS), drives the learning network process individually in each dataset so that results are pooled into a single network. The group network is usually formed by including the edges that exist for most subjects. An example of this approach is Oates (2013) who proposed an algorithm that first scored individuals and then constructed a“group network” by minimising the distance between the individual and this group network. Although the IS approach seems to cope well with the different interactions, its results are often inconsistent amongst individuals because subjects tend in practice to display obvious heterogeneities Gonçalves et al. (2001), Mechelli et al. (2002). We show an example of this in Section 4.

It is not possible to say which method is generally superior over the others since the interpretation of results depends directly on the assumptions of each method which can vary across different populations and experiments (Li et al., 2008). However, it is fair to say that although some of these methods explicitly recognise the intra-subject variability, none of them assesses the homogeneity of the individual connectivity maps. If the approaches described above are applied to a heterogeneous group, then any conclusions based on the group network can therefore obviously be misleading. A fourth method, the *group-structure* (GS) approach, aims to model such potential heterogeneity between subjects.

The GS approach studies group homogeneity through cluster analysis, considering a particular measure of similarity between subjects. If this analysis suggests that subjects should be clustered into disjoint subgroups, then this group of subjects is not homogeneous. When heterogeneity is found to be present, it is suggested that any subsequent analyses of interest should be done for every subgroup independently. Note that no prior classification information is necessary to use these methods. For instance, Kherif et al. (2004) defined a separation measure between two subjects’ data based on a multivariate correlation. In another example, Gates (2012) estimated the effective connectivity of both individual and group network using the Group Iterative Multiple model estimation (GIMME; Gates and Molenaar, 2012). In this work, Gates (2012) proposed the correlation between connectivity strengths as the separation measure between subjects. In this paper, we propose an alternative separation measure between subjects as a function of the model selection criterion: the Bayes factor.

## 3 Group analysis using the multiregression dynamic model

In this section we first describe MDM, a Bayesian dynamic regression model used to estimate effective connectivity. Then we show how VTS, the CS and the IS approaches can be applied in the context of the MDM. Finally we present a novel GS approach that uses a distance based on one used for a model selection criterion.

### 3.1 The multiregression dynamic model

MDM is a class of multivariate time series models which embeds putative causal hypotheses among its variables over time Queen and Smith (1993), Queen and Albers (2009). In MDM, the multivariate model for observable series $Y_{t}\left(1\right),\ldots ,Y_{t}\left(n\right)$ is broken down into simpler univariate regression dynamic linear models (DLMs) so that the effective connectivity (or parents’ effect of one node) is allowed to vary across the period of an experiment. Formally, this model is described by the following n observation equations, system equation and initial information (Queen and Smith, 1993):

$$\mathrm{Observation equations :}Y_{t}\left(r\right)∼N\left(F_{t}{\left(r\right)}^{\prime }\theta_{t}\left(r\right),V\left(r\right)\right);\;\;\;\;\;\;\;\;\;\;\;\;\;\;\;\;\;\;\;\;\;\;\;\;\;\;\;\;\;\;\;\;$$

$$\mathrm{System equation :}\theta_{t}∼N\left(\theta_{t-1},W_{t}\right);\;\;\;\;\;\;\;\;\;\;\;\;\;\;\;\;\;\;\;\;\;\;\;\;\;\;\;\;\;\;\;\;\;\;\;\;\;\;\;$$

where r=1,…,n regions, t=1,…,T time points, N denotes the Gaussian distribution, $\theta_{t}^{\prime }=\left(\theta_{t}{\left(1\right)}^{\prime },\ldots ,\theta_{t}{\left(n\right)}^{\prime }\right)$, $\theta_{t}{\left(r\right)}^{\prime }$ is the $p_{r}$-dimensional parameter vector for $Y_{t}\left(r\right)$ and $F_{t}\left(r\right)$ is a known linear function of the parents Pa(r) and assumed fixed. For nodes that do not have parents, $F_{t}\left(r\right)=1$. In addition, $W_{t}\left(r\right)$ are $p_{r}$ square matrices which form $W_{t}=\text{blockdiag}\left\{W_{t}\left(1\right),\ldots ,W_{t}\left(n\right)\right\}$.

The initial information is defined as follows:

$$\left(\theta_{0}|y_{0}\right)∼N\left(m_{0},C_{0}\right);$$

$$\left(\phi \left(r\right)|y_{0}\right)∼G\left(\frac{n_{0}\left(r\right)}{2},\frac{d_{0}\left(r\right)}{2}\right);$$

where ϕ(r) is the observation precision defined as $V{\left(r\right)}^{-1}$ and G represents the Gamma distribution given density explicitly. The initial information is the probabilistic representation of the previous knowledge about θ given the information at time t=0, *i.e.* $y_{0}$. The mean vector $m_{0}$ is an initial estimate of the parameters whilst the variance–covariance matrix $C_{0}$ represents the uncertainty about this mean and the relationship among the parameters. $C_{0}\left(r\right)$ is $p_{r}$ square matrix and is defined as $C_{0}=\text{blockdiag}\left\{C_{0}\left(1\right),\ldots ,C_{0}\left(n\right)\right\}$. Because the equations of the MDM can be viewed as a collection of nested univariate DLMs, the parameters can be estimated using well-known Kalman Filter recurrences over time. Therefore, as the relationship between the observed variables and their parents is linear, the errors follow a Gaussian distribution whilst the precision has a Gamma distribution, these lead to a conjugate analysis thus ensure a parametric economy and identifiability of the model and perhaps most potently extremely first formulated for evaluating the fit of any potential connectivity group.

Thus one of the most popular ways of comparing two models is to use the Bayes factor measure Jeffreys (1961), West and Harrison (1997). This is defined as the ratio between the predictive likelihood of two models, model $m_{0}$ and model $m_{1}$, say. The joint log predictive likelihood (LPL) is calculated as

(1) $$\text{LPL}\left(m\right)=\overset{n}{\underset{r=1}{\sum }}\overset{T}{\underset{t=1}{\sum }}\log p\left(y_{t}\left(r\right)|y^{t-1},Pa\left(r\right),m\right),$$

where $y^{t-1}=\left(y_{1},\ldots ,y_{t-1}\right)$ is the observed value of time series until time t−1, the conditional forecast distribution has the closed form of a student’s t-distribution and m reflects the current choice of model that determines the relationship between n regions (Costa et al., 2015). To compare model $m_{1}$ to model $m_{0}$, we use the log Bayes factor (BF) so that

$$\log\text{(BF)}=\text{LPL}\left(m_{1}\right)-\text{LPL}\left(m_{0}\right).$$

Heuristically the higher the score of a model the better its fit to the observed data.

To search over networks we have used the recent MDM-IP algorithm (Costa et al., 2015) which is an efficient search-and-score method using scores found through Eq. (1), the *gobnilp* system Cussens (2011), Bartlett and Cussen (2013) and the SCIP IP framework (Achterberg, 2007). In our case, for any candidate model m, LPL(m) is a sum of n ‘local scores’, one for each node r, each corresponding to various scores of models hypothesising a causal relationship from other nodes. The local score for $Y_{t}\left(r\right)$ is thus determined by the choice of parent set $Pa_{m}\left(r\right)$ specified by the model m. Let $c\left(r,Pa_{m}\left(r\right)\right)$ denote this local score, so that $\mathrm{LPL}\left(m\right)=\sum_{r=1}^{n}c\left(r,Pa_{m}\left(r\right)\right)$. We can now view a model selection for the MDM as a search for n subsets Pa(1),…,Pa(n) which maximise the LPL(m) subject to existing an MDM model m, with $Pa\left(r\right)=Pa_{m}\left(r\right)$ for r=1,…,n, corresponding to the best search set of causal relationships under the constraint that the whole system must be acyclic (see details in Costa et al., 2015). It is also possible to search for graphical structure without the constraints of DAG, choosing the set of parents that maximise the LPL for each node independently. We called this algorithm the MDM-DGM (Directed Graph Model; Costa et al., 2017).

### 3.2 Some approaches for group analysis

The VTS approach finds a typical subject, assuming the same network with exactly the same connectivity for all subjects. Within an MDM framework, the “typical subject” was defined by first calculating the average of time series variables over all subjects. Based only on this “ordinary subject”, the search method as applied in Costa et al. (2015) can then be used to find the group network. Note that the local score for node r can now be written as follows:

$$c_{a}\left(r,\overset{¯}{M}\left(r\right)\right)=\overset{T}{\underset{t=1}{\sum }}\log p\left({\bar{y}}_{t}\left(r\right)|{\bar{y}}^{t-1},{\bar{x}}_{t}\left(r\right),\overset{¯}{M}\left(r\right)\right),$$

where ${\bar{y}}_{t}\left(r\right)$ is the average of observed variables at time t and node r over subjects, ${\bar{y}}^{t}={\left({\bar{y}}_{1},\ldots ,{\bar{y}}_{t}\right)}^{\prime }$, ${\bar{y}}_{t}={\left({\bar{y}}_{t}\left(1\right),\ldots ,{\bar{y}}_{t}\left(n\right)\right)}^{\prime }$, and ${\bar{x}}_{t}\left(r\right)={\left({\bar{y}}_{t}\left(1\right),\ldots ,{\bar{y}}_{t}\left(r-1\right)\right)}^{\prime }$. Here $\overset{¯}{M}\left(r\right)$ is the model defined by the parent set of node r so that the group network consists of $\overset{¯}{M}=\left(\overset{¯}{M}\left(1\right),\ldots ,\overset{¯}{M}\left(n\right)\right)$. The connectivity strength for the group network is estimated based on the smoothed posterior distribution of parameters θ’s using the MDM fitted for each such typical subject.

The CS approach assumes that all subjects share the same group network structure, but that the parameters may differ over subjects. In this way, the parameter estimation process can be applied to each subject independently. Therefore for individual models represented by the same graph $\overset{¯}{M}$, the scores used in search process are defined as follows:

(2) $$c\left(r,\overset{¯}{M}\left(r\right)\right)=\overset{S}{\underset{i=1}{\sum }}\overset{T}{\underset{t=1}{\sum }}\log p\left(y_{it}\left(r\right)|y_{i}^{t-1},x_{it}\left(r\right),\overset{¯}{M}\left(r\right)\right),$$

where S is the number of subjects, $y_{it}\left(r\right)$ is the observed variable for region r and subject i at time t, $y_{i}^{t-1}$ is the observed cumulative data until time t−1 for subject i, and $x_{it}\left(r\right)={\left(y_{it}\left(1\right),\ldots ,y_{it}\left(r-1\right)\right)}^{\prime }$.

Note that within the CS approach the parents of a particular node r are the same for all subjects ($\overset{¯}{M}\left(r\right)$). The group network is estimated using a search algorithm whose scores are given in Eq. (2). The connectivity strength for group network is estimated as the average of the smoothed estimates of θ’s over subjects.

In contrast, using this model the IS approach usually learns individual networks independently, using the individual scores

(3) $$c_{i}\left(r,M_{i}\left(r\right)\right)=\overset{T}{\underset{t=1}{\sum }}\log p\left(y_{it}\left(r\right)|y_{i}^{t-1},x_{it}\left(r\right),M_{i}\left(r\right)\right),$$

where $M_{i}\left(r\right)$ is the model defined by the parent set of node r for subject i so that the individual network for subject i consists of $M_{i}=\left(M_{i}\left(1\right),\ldots ,M_{i}\left(n\right)\right)$. The group network structure ($\overset{¯}{M}$) consists of the edges that exist in the individual network for most subjects. MDM is then fitted for all subjects using the group network and, as in the CS approach, the connectivity strength is estimated as the average of the smoothed estimates of θ’s over subjects.

### 3.3 Clustering with pairwise log Bayes factor separation

Here we define a new method designed to be sensitive to potential heterogeneities over the connectivity graphs of different subjects as well as their connectivity strengths. In this group-structure (GS) approach subjects are first grouped according to the similarities in their graphs. These similarities are defined by a separation measure, d(i,j), calculated for every pair of subjects i and j. The individual networks, $M_{i}$, with the pairwise group network, $m_{G}$, are then compared using

$$d\left(i,j\right)=c_{ij}\left(m_{I}\right)-c_{ij}\left(m_{G}\right),$$

where $m_{I}=\left(M_{i},M_{j}\right)$,

$$c_{ij}\left(m_{I}\right)=\overset{n}{\underset{r=1}{\sum }}\left[c_{i}\left(r,M_{i}\left(r\right)\right)+c_{j}\left(r,M_{j}\left(r\right)\right)\right],$$

$$c_{ij}\left(m_{G}\right)=\overset{n}{\underset{r=1}{\sum }}\left[c_{i}\left(r,m_{G}\left(r\right)\right)+c_{j}\left(r,m_{G}\left(r\right)\right)\right],$$

$m_{G}=\left(m_{G}\left(1\right),\ldots ,m_{G}\left(n\right)\right)$, for i∈{1,…,S−1}, j∈{2,…,S}, j>i. So here the individual networks, $M_{i}$, are estimated by maximising the scores in Eq. (3). The pairwise group networks, $m_{G}$, is then estimated by maximising the sum of scores for only two subjects, i and j, such as in Eq. (2), considering $\overset{¯}{M}\left(r\right)=m_{G}\left(r\right)$.

Some properties of d(i,j) are given below.

1. For the MDM-IPA, the scores are exactly the LPL. So d(i,j) can be seen as the logBF comparing the model that assumes subjects i and j have different graphical structures to one where it is assumed that they share the same one. Thus, we call this separation measure *the pairwise logBF separation*.
2. The pairwise logBF separation is symmetric, *i.e.* d(i,j)=d(j,i).
3. If the estimated individual graphical structures for subjects i and j are the same, then d(i,j)=0. As $M_{i}$ is the network that maximises the scores in Eq. (3), then $c_{i}\left(M_{i}\right)>c_{i}\left(M_{i}^{*}\right)$, where $c_{i}\left(M_{i}\right)=\sum_{r=1}^{n}c_{i}\left(r,M_{i}\left(r\right)\right)$ and $M_{i}^{*}$ is any possible network for subject i, except $M_{i}$. Thus,
  $$c_{i}\left(M_{i}\right)+c_{j}\left(M_{j}\right)>c_{i}\left(M_{i}^{*}\right)+c_{j}\left(M_{j}^{*}\right).$$
  By definition, the pairwise group network assumes that both subjects share the same graphical structure, *i.e.* $M_{i}^{*}=M_{j}^{*}=m_{G}^{*}$. As a result, when $M_{i}=M_{j}$, the above inequality becomes
  $$c_{i}\left(M_{i}\right)+c_{j}\left(M_{i}\right)>c_{i}\left(M_{i}^{*}\right)+c_{j}\left(M_{i}^{*}\right)$$
  (4) $$c_{ij}\left(M_{i}\right)>c_{ij}\left(m_{G}^{*}\right).$$
  Because, by definition, $m_{G}^{*}$ is a network different from $M_{i}$ and given Eq. (4), $M_{i}$ is the pairwise group network that maximises the scores in Eq. (2), *i.e.* $m_{G}=M_{i}$. Therefore,
  $$d\left(i,j\right)=\overset{n}{\underset{r=1}{\sum }}\left(c_{i}\left(r,M_{i}\left(r\right)\right)+c_{j}\left(r,M_{j}\left(r\right)\right)\right)-c_{ij}\left(m_{G}\right),$$
  $$\text{ as }M_{i}=M_{j}\text{, then }d\left(i,j\right)=c_{ij}\left(M_{i}\right)-c_{ij}\left(m_{G}\right),$$
  $$\text{ and as }M_{i}=m_{G}\text{, then }d\left(i,j\right)=0.$$
4. By definition, the separation d(i,j) is non-negative. That is, because $c_{k}\left(M_{k}\right)\geq c_{k}\left(M_{k}^{*}\right)$, whenever $M_{k}$ is selected by maximising the scores in Eq. (3), and $M_{k}^{*}$ is any possible individual network for subject k for k=i,j. Thus,
  $$c_{i}\left(M_{i}\right)+c_{j}\left(M_{j}\right)\geq c_{i}\left(M_{i}^{*}\right)+c_{j}\left(M_{j}^{*}\right)$$
  $$\text{letting }M_{i}^{*}=M_{j}^{*}=m_{G}\text{, }c_{i}\left(M_{i}\right)+c_{j}\left(M_{j}\right)\geq c_{ij}\left(m_{G}\right)$$
  $$c_{ij}\left(m_{I}\right)-c_{ij}\left(m_{G}\right)\geq 0$$
  d(i,j)≥0.

Using a cluster analysis with these pairwise logBF separations, subjects are grouped according to their similar networks. Then Eq. (2) is used to score models for subjects belonging to the same subgroup. As a result a graphical structure is estimated for each one of subgroups ${\overset{¯}{M}}_{1},\ldots ,{\overset{¯}{M}}_{G}$ independently, where G is the number of subgroups. Only then is the connectivity strength estimated per subgroup as the average of estimated parameters θ’s over (the approximately homogeneous) subjects belonging to the same subgroup.

To compare the computational cost of these methods recall that learning the individual network follows two basic steps: (1) calculating the scores for each set of parents for individual nodes, (2) calculating the optimal MDM using the MDM-IPA or the MDM-DGM over the full model. The run-time of the first step depends critically on the number of nodes and the sample size. It is necessary to fit a linear dynamic model for every node and every set of parents — there are $2^{n-1}$ possible sets of parents per node. We have found that step 1 takes dramatically longer time than the step 2, see Costa et al. (2015). For example, for an 11-node networks with 100 time-points, step 1 took around 168 min whilst step 2 took around 30 s, on a 2.7 GHz quad-core Intel Core i7 Linux host with 16 GB. Thus, the VTS approach takes the shortest time because the search network algorithm (steps 1 and 2) is applied only once for a “typical” subject. In contrast, in the CS approach, step 1 is applied for each subject and then the scores are summed over all individuals whilst step 2 is applied only once. For the IS and GS approaches, steps 1 and 2 are applied individually for all subjects. Therefore, as step 2 only takes a relatively short time, the difference between the IS and CS tends to be small. However, as individual scores are summed before step 2 can be applied for every pair of subjects, the GS approach takes longest. The greater the number of subjects the bigger is these run time differences.

## 4 Comparing methods using synthetic data

In this section, we compare the four group analysis approaches described above using synthetic data. The aim of this section is to assess the efficacy of methods when subjects are sampled from populations whose individuals may exhibit different networks. In order to obtain more realistic data, we simulated data based on the results found in the analysis with real datasets and showed in Section 5. Therefore, we considered 3 different DAGs that were estimated for most people in real situation (subgroups 1, 2 and 4 from Fig. 5), and we repeat DAGs here in Fig. 1 (DAG1, DAG2 and DAG3) for easy viewing. We simulated data for 10 subjects for each DAG, considering 12 nodes, 1158 time points and true parameters as the average of estimates over subjects found in Section 5.

*The GS approach*

**Fig. 2:**
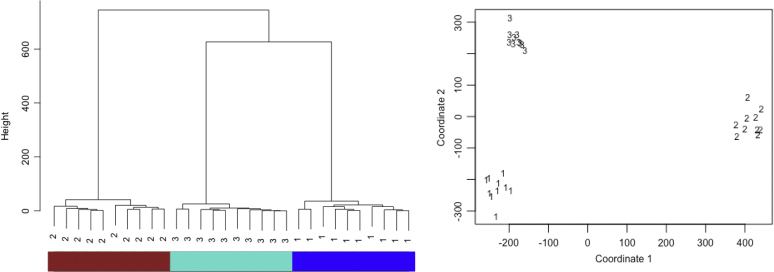
Dendrogram (*left*) and MDS (*right*) for synthetic data using the pairwise logBF separation. Numbers 1, 2 and 3 correspond to subjects simulated based on DAG1, DAG2 and DAG3, respectively. Coloured rectangles under the dendrogram identify the three subgroups found by the hybrid algorithm. (For interpretation of the references to colour in this figure legend, the reader is referred to the web version of this article.)

The pairwise logBF separation for all pairs of subjects was evaluated as shown in Section 3.3 and considering the MDM-IPA. In order to assess the homogeneity of this group, we used *the hierarchical cluster* ( Everitt et al., 2011, Chapter 4) and the *multidimensional scaling* (MDS; Everitt et al., 2011, Section 2.3.3). The hierarchical cluster results can be illustrated through a *dendrogram* and, to define subgroups, we use the *dynamic tree cut* (hybrid algorithm; Langfelder et al., 2008). Fig. 2 (left) shows the dendrogram which was found using the R packages *hclust* and *dynamicTreeCut*, and the minimum size of cluster equal to 3. In this diagram, subjects are represented by the indexing of their respective DAG. The hybrid algorithm correctly identifies the number of subgroups, *i.e.* the three coloured rectangles under the dendrogram and all subjects were correctly grouped. Moreover, the average separation between subjects belonging to the same group was around 13 whilst the average separation between groups was almost 603. This provides strong evidence that people from different subgroups have different graphical structures.

Following this, multidimensional scaling (MDS) was explored in this context. MDS depicts patterns in the separation between subjects with a Euclidean space. By using geometry, the best separation between subjects in a low-dimensional scaling is used to represent the original dissimilarity measure ( Everitt et al., 2011, Section 2.3.3). Note that, from this MDS plot, it is possible to recognise subgroups and outliers, and also to verify a measure of the quality of this approximate Euclidean depiction. For instance, Fig. 2 (right) shows a 2D plot which captured almost 99% of this information, where subjects are labelled according to the number of their DAG as before. Clearly subjects from DAG1 are on the left and bottom of the figure, whilst subjects from DAG2 are on right and DAG3 on the left and top.

*Comparing group analysis approaches*

The graphical structures for the VTS, CS and IS approaches were estimated as described in Section 3.2, considering the MDM-IPA (see Fig. 1). As there are three true DAGS and as these approaches only estimated a single DAG, we considered a graph formed by 24 edges that exist for most people (more than 2∕3 of individuals) in order to compare the performance of methods. Also we estimated *sensitivity* and *specificity* measures in which the former represents the proportion of edges that exist in the true graph and are correctly identified as such in the estimated graph, whilst the latter represents the proportion of edges that do not exist in the true graph that are correctly identified as such in the estimated graph. Table 1 shows these estimated measures for all four approaches.

Unsurprisingly the IS approach picked up the edges that exist for most individuals, *i.e.* 87.5% of these popular 24 edges, and estimated only more 5 false-positive edges. The VTS approach provides the worst performance, estimating only 58% of popular edges and more 29 false-positive edges — the averaging of the time series over subjects resulted a bad estimated graph in some way.

In contrast to other methods, the GS approach identified the three different networks perfectly, that were made up of 10 individuals in each one (see Fig. 2). As a result the estimated sensitivity and specificity were one of the highest measures among the methods, with the average of 87% and 95% for these measures, respectively, as shown in Table 1.

**Table 1:** The estimated sensitivity and specificity measures for VTS, CS, IS and GS approaches. These measures were estimated for the first 3 approaches considering a graph formed by most popular edges whilst all true DAGs were considered for the GS approach.

Approach	Sensitivity	Specificity
VTS	58%	73%
CS	71%	87%
IS	88%	95%
GS-subgroup 1	83%	94%
GS-subgroup 2	96%	96%
GS-subgroup 3	83%	95%

## 5 Group-structure in practice

**Fig. 3:**
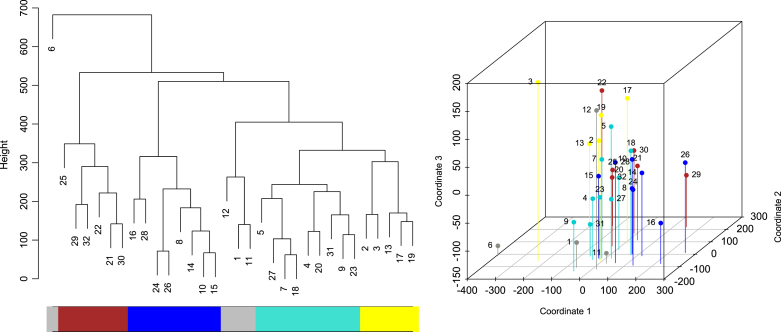
Dendrogram (*left*) and MDS (*right*) of real fMRI data using the pairwise logBF separation for the MDM-IPA. Coloured rectangles under dendrogram identify subgroups found by the hybrid algorithm. Subgroup 1 is defined as Red; Subgroup 2 is Royal Blue; Subgroup 3 is Grey (without subject 6); Subgroup 4 is Light Blue; Subgroup 5 is Yellow; and Subgroup 6 is Subject 6. The MDS graph illustrates the subjects with respective colours, and captured almost 75% of the information provided by the dissimilarity measure. (For interpretation of the references to colour in this figure legend, the reader is referred to the web version of this article.)

This real application consists of a resting-state fMRI data setting from a 15-min experiment with 32 subjects (TR = 1140 ms, 2 × 2 × 2 mm). ROIs were defined either functionally or based on the Harvard-Oxford atlas: left and right amygdala, ventromedial prefrontal cortex (VMPFC), left and right dorsolateral prefrontal cortex (DLPFC), left and right posterior insula (PostInsula), left and right anterior insula (AntInsula), left and right orbitofrontal cortex (OFC), and anterior midcingulate cortex (aMCC). More details can be found in Bijsterbosch et al. (2015). The GS approach was applied and the result can be seen in Fig. 3 through a dendrogram (*left*) and the MDS plot (*right*), considering the MDM-IPA. Based on the hybrid algorithm, we defined six subgroups: red as subgroup 1; royal blue as subgroup 2; grey (without subject 6) as subgroup 3; light blue as subgroup 4; yellow as subgroup 5; and Subject 6 as subgroup 6.

**Fig. 4:**
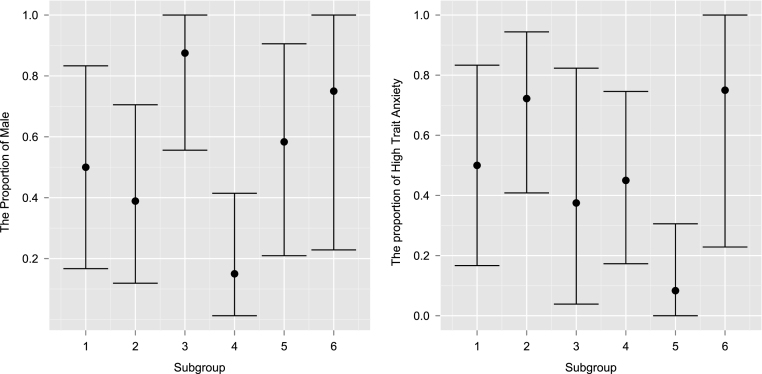
The proportion of male (left) and the proportion of subjects who have high trait anxiety (right) by subgroup defined in Fig. 3. The bars represent 95% HPD interval calculated assuming a non-informative prior.

It is possible to use some characteristics of the subjects to begin to explain the differences between subgroups. For example, Fig. 4 shows a significant difference in the percentage of men between subgroup 3 and 4 (*left*), and in the percentage of high trait anxiety individuals between subgroup 2 and 5.

**Fig. 5:**
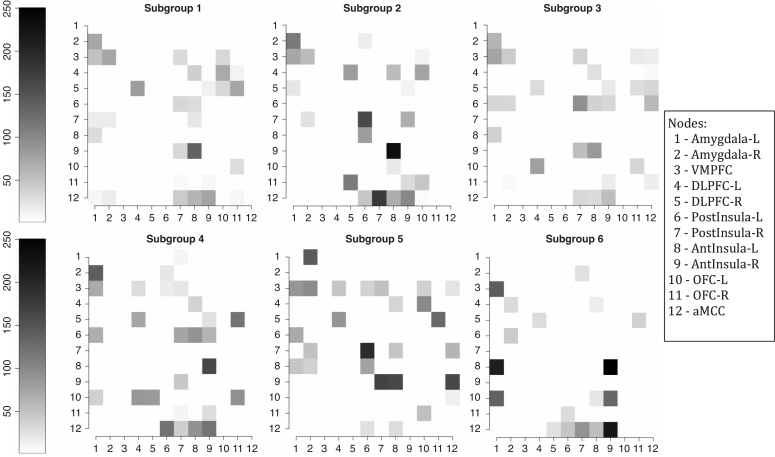
The connectivity strength standardised mean (${\overset{¯}{Z}}_{ig}$) for a particular edge i→j, where i indexes rows and j columns, using the *MDM-IPA* per subgroup defined in Fig. 3, only for significant connectivities, *i.e.*${\overset{¯}{Z}}_{ig}\geq 2$.

The graphical structures for each subgroup were estimated independently, summing the scores of subjects belonging to the same subgroup and then applying the MDM-IPA. To simplify the analysis, we calculated the connectivity strength standardised mean as ${\overset{¯}{Z}}_{ig}=\frac{{\overset{¯}{\theta }}_{ig}}{\sqrt{{\overset{¯}{\sigma^{2}}}_{ig}∕TS_{g}}}$, where ${\overset{¯}{\theta }}_{ig}$ and ${\overset{¯}{\sigma^{2}}}_{ig}$ are the average of location and scale parameters of the smoothed distribution over time and subjects for connectivity i and subgroup g; T is the sample size; and $S_{g}$ is the number of subjects in the subgroup g. Fig. 5 provides the connectivity strength standardised mean for a particular edge i→j, where i indexes rows and j columns, per subgroup and only for those edges with significant Binomial tests after false discovery rate correction (FDR; Benjamini and Hochberg, 1995). We noted that ${\overset{¯}{Z}}_{ig}\approx 458$ for connectivity from node 8, AntInsula-L, to node 9, AntInsula-R, in subgroup 6, but we attributed the value 250 in this figure for clarifying the differences among other connectivities.

**Fig. 6:**
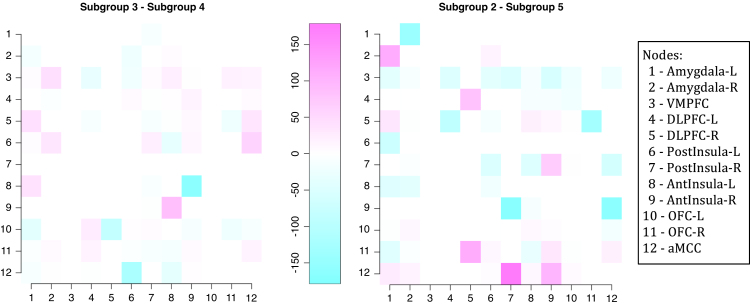
The connectivity strength standardised difference (${\overset{¯}{D}}_{igl}$) for a particular edge i→j, where i indexes rows and j columns, between subgroup 3 and subgroup 4 (*left*), and between subgroup 2 and subgroup 5 (*right*). (For interpretation of the references to colour in this figure legend, the reader is referred to the web version of this article.)

Note that not only the existence of the effective connectivity differs among subgroups, but also the connectivity strength may vary from one subgroup to other. For instance, suppose we are interested in comparing subgroup 3 to subgroup 4 and subgroup 2 to subgroup 5, because they appear to have a correlation with covariates gender and high trait anxiety, as shown in Fig. 4. Then we could argue we should calculate the connectivity strength standardised difference as ${\overset{¯}{D}}_{igl}=\frac{{\overset{¯}{\theta }}_{ig}{\overset{¯}{\theta }}_{il}}{\sqrt{{\overset{¯}{\sigma^{2}}}_{ig}∕TS_{g}+{\overset{¯}{\sigma^{2}}}_{il}∕TS_{l}}}$ as a coarse measure of connectivity i in subgroup g compared to subgroup l. Fig. 6 (*left*) provides this difference in connectivity strength between subgroups 3 and 4, in which the percentage of male is higher in the former subgroup than the latter one (Fig. 4 *left*). Pink connectivities mean they are stronger in subgroup 3 (men) than in subgroup 4 (women), whilst blue connectivities are stronger in subgroup 4 (women) than in the subgroup (men). Note that the connectivity from node 8 (AntInsula left) to node 9 (AntInsula right) is the strongest in subgroup 4 (women) whilst the reciprocal connectivity, from node 9 to 8, is the strongest in subgroup 3 (men). Also connectivities in which the parent is node 12 (aMCC) tend to be stronger in subgroup 4 (women) than in the male subgroup.

Another example is the difference between subgroups 2 and 5 where the proportion of subjects who have high trait anxiety is larger in the former subgroup than latter one (Fig. 4 *right*). The connectivity strength standardised difference (${\overset{¯}{D}}_{i25}$) between these two subgroups can be seen in Fig. 6 (*right*). An interesting result is that some connectivities are the strongest in one subgroup whilst their *reciprocal* connectivities are the strongest in other subgroup. For instance, the connectivity from node 1 (left Amygdala) to node 2 (right Amygdala) is the strongest in subgroup 5 whilst the reciprocal connectivity, from node 2 to 1, is the strongest in subgroup 2. A similar result can be seen between the following pairs of nodes: 4 and 5 (left and right DLPFC); 5 and 11 (right DLPFC and right OFC); 7 and 9 (right postInsula and right antInsula); 7 and 12 (right postInsula and aMCC); 9 and 12 (right antInsula and aMCC). Note that most of these connectivities are on the right side of the brain.

## 6 The estimation of multiple networks

We discussed above four approaches which are used to estimate a group network, considering the different ways of combining individual information. In this section, we consider a complementary problem and provide ways to infer all graphs: individual, subgroup and group networks. We present the following two methods here: the *Individual Estimation of Multiple Networks* (IEMN) and the *Marginal Estimation of Multiple Networks* (MEMN). All these methods incorporate the GS approach to deal with heterogeneous group, but the second one, MEMN, appears to be more robust than IEMN, because it estimates the individual network using information from other subjects.

In Section 6.1, we define the method IEMN in which subjects are first grouped using a cluster analysis, as in the GS approach. Then, IEMN first estimates the individual networks independently, and after that, the subgroup and the group networks are estimated using the CS approach. This method therefore addresses mainly the challenge about the heterogeneous population problem. The second method, MEMN, is then developed based on a method suggested by Oates (2013), in which subgroup and group networks are estimated using the IS approach and a similarity measure between individual and subgroup/group network structures. This method then estimates the individual networks using the information of other subjects belonging to the same homogeneous subgroup. IEMN and MEMN are compared using real fMRI data in Section 6.2.

### 6.1 The IEMN and the MEMN

In this section we describe a new approach for searching over MDMs which not only estimates group networks but also individual networks, whilst taking into account the information from other subjects. This approach, called the Marginal Estimation of Multiple Networks (MEMN), was originally developed for Dynamic Bayesian Networks (DBNs), using a penalty function that represents the distance between group and individual networks (Oates, 2013). We generalise this method by combining it with the GS approach, *i.e.* the cluster analysis shown above, including one more step to estimate the subgroup networks. Oates (2013) used his method to consider the probability of a particular edge existing. Here, in contrast, we develop MEMN based on the log predictive likelihood (LPL). Furthermore, Oates (2013) assumed that the parameter of the penalty function, λ, was known, being defined by scientists. It may not be easy to suggest appropriate values for this parameter, especially when the study consists of a novel experiment, and a misspecification of this parameter will provide erroneous results. We discuss here some new possibilities for estimating λ from data, for example, minimising LPL.

A comparison between the MEMN and the Individual Estimation of Multiple Networks (IEMN) is also provided in this section. The IEMN is basically the GS approach described above, where the individual networks were estimated independently whilst the subgroup networks were estimated summing the scores over subjects within the same subgroup.

**Fig. 7:**
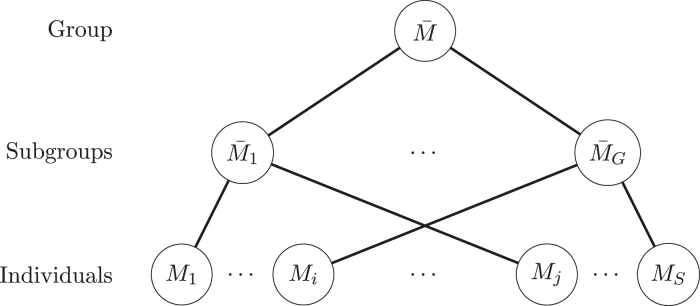
Individual networks: $M_{1},\ldots ,M_{S}$; Subgroup networks: ${\overset{¯}{M}}_{1},\ldots ,{\overset{¯}{M}}_{G}$, found by cluster analysis; and the group network: $\overset{¯}{M}$.

Reviewing the notation, $M_{i}$ is a graphical structure within the space $M_{i}$ for subject i; ${\overset{¯}{M}}_{g}$ is a graphical structure within the space ${\overset{¯}{M}}_{g}$ of subgroup g=1,…,G. $S_{g}$ is the set of the indices of subjects belonging to the subgroup g, according to cluster analysis, and $S_{g}$ denotes the number of subjects in the subgroup g. $\overset{¯}{M}$ denotes a graphical structure of the group considering all subjects within the space $\overset{¯}{M}$ (see Fig. 7).

#### 6.1.1 The Individual Estimation of Multiple Networks (IEMN)

*IEMN: Individual Graphical Structures:* $M_{1},\ldots ,M_{S}$

The maximum a posteriori probability (MAP) estimator of $M_{i}$ for IEMN can be defined as

$${\overset{ˆ}{M}}_{i}≔{arg max}_{M_{i}\in M_{i}}p\left(y_{i}^{T}|M_{i}\right),$$

where $\log p\left(y_{i}^{T}|M_{i}\right)=\sum_{r=1}^{n}c_{i}\left(r,M_{i}\left(r\right)\right)$. The score $c_{i}\left(r,M_{i}\left(r\right)\right)$ is found per subject i and node r as in Eq. (3). Therefore the MDM-IPA or the MDM-DGM can be applied to find ${\overset{ˆ}{M}}_{i}$, per subject independently, using the scores $c_{i}\left(r,M_{i}\left(r\right)\right)$.

*IEMN: Subgroup Graphical Structures:* ${\overset{¯}{M}}_{1},\ldots ,{\overset{¯}{M}}_{G}$

Now the MAP estimator of the subgroup network ${\overset{¯}{M}}_{g}$ is:

$${\overset{ˆ}{\overset{¯}{M}}}_{g}≔{arg max}_{{\overset{¯}{M}}_{g}\in {\overset{¯}{M}}_{g}}\overset{n}{\underset{r=1}{\sum }}c\left(r,{\overset{¯}{M}}_{g}\right),$$

where $c\left(r,{\overset{¯}{M}}_{g}\right)$ is defined as in Eq. (2) for $i\in S_{g}$. Then ${\overset{¯}{M}}_{g}$ can be estimated by MDM-IPA or MDM-DGM per subgroup independently.

*IEMN: Group Graphical Structure:* $\overset{¯}{M}$

To search the group network, the CS approach can be applied so that the scores are summed over all subjects, as in Eq. (2). Then the MAP estimator of the group network for the whole population can be defined as

$$\overset{ˆ}{\overset{¯}{M}}≔{arg max}_{\overset{¯}{M}\in \overset{¯}{M}}p\left(y|\overset{¯}{M}\right),$$

where $\log p\left(y|\overset{¯}{M}\right)=\sum_{r=1}^{n}c\left(r,\overset{¯}{M}\left(r\right)\right)$, and $y^{\prime }=\left(y_{1}^{T^{\prime }},\ldots ,y_{S}^{T^{\prime }}\right)$. Again $\overset{¯}{M}$ can be estimated by the MDM-IPA or the MDM-DGM.

#### 6.1.2 The Marginal Estimation of Multiple Networks (MEMN)

*MEMN: Individual Graphical Structures:* $M_{1},\ldots ,M_{S}$

This method scores individual networks based on the information from the subgroup network and the individual networks of other subjects who belong to the same subgroup, as follows (see details in Appendix B).

(5) $$p\left(M_{i}\left(r\right)|y\right)=\underset{{\overset{¯}{M}}_{g}\left(r\right)\in {\overset{¯}{M}}_{g}\left(r\right)}{\sum }\left[p\left(y_{i}^{T}|M_{i}\left(r\right)\right)\times p\left(M_{i}\left(r\right)|{\overset{¯}{M}}_{g}\left(r\right)\right)\times \;\underset{k\in S_{g}\setminus \left\{i\right\}}{\prod }\underset{M_{k}\left(r\right)\in M_{k}\left(r\right)}{\sum }\left(p\left(y_{k}^{T}|M_{k}\left(r\right)\right)\times p\left(M_{k}\left(r\right)|{\overset{¯}{M}}_{g}\left(r\right)\right)\right)\right],$$

where a subject i belongs to the subgroup g; $k=1^{*},\ldots ,S_{g}^{*}$ whenever the kth element of $S_{g}$; and $S_{g}\setminus \left\{i\right\}$ is the set of the indexes of all subjects belonging to the subgroup g, except for subject i. Here ${\overset{¯}{M}}_{g}=\left({\overset{¯}{M}}_{g}\left(1\right),\ldots ,{\overset{¯}{M}}_{g}\left(n\right)\right)$; and $M_{i}=\left(M_{i}\left(1\right),\ldots ,M_{i}\left(n\right)\right)$. Note that the term $p\left(y_{i}^{T}|M_{i}\left(r\right)\right)=\exp\left\{c_{i}\left(r,M_{i}\left(r\right)\right)\right\}$, from Eq. (3), for i=1,…,S; and the other term is defined as follows:

$$p\left(M_{i}\left(r\right)|{\overset{¯}{M}}_{g}\left(r\right)\right)\propto \exp\left\{-\lambda_{irg}d_{irg}\right\},$$

where $d_{irg}$ is the Structural Hamming Distance (SHD) between $M_{i}\left(r\right)$ and ${\overset{¯}{M}}_{g}\left(r\right)$, *i.e.* the number of nodes that are the parents of node r only in one network: $M_{i}\left(r\right)$ or ${\overset{¯}{M}}_{g}\left(r\right)$. We reduce the number of hyperparameters by assuming that the parents of node r are a priori equally likely to be shared between the subject i and subgroup g, *i.e.* $\lambda_{irg}=\lambda$ for all i, r, and g. The hyperparameter λ is usually specified in a subjective manner. Oates (2013) suggested writing λ as a function of the probability of maintaining the status (present/absent) of the edge j between the individual network $M_{i}$ and the subgroup network ${\overset{¯}{M}}_{g}$. That is,

(6) $$p\left(j\notin M_{i}\Delta {\overset{¯}{M}}_{g}\right)=\frac{\exp\left\{-\lambda \times 0\right\}}{\exp\left\{-\lambda \times 0\right\}+\exp\left\{-\lambda \times 1\right\}}=\frac{1}{1+\exp\left\{-\lambda \right\}}\text{.}$$

Here AΔB denotes the set of elements contained in A but not in B plus elements contained in B but not in A. Therefore, the odds of an individual graph are the same as its subgroup graph regarding a particular edge

$$O_{\lambda }≔\frac{p\left(j\notin M_{i}\Delta {\overset{¯}{M}}_{g}\right)}{p\left(j\in M_{i}\Delta {\overset{¯}{M}}_{g}\right)}=\frac{\exp\left\{-\lambda \times 0\right\}}{\exp\left\{-\lambda \times 1\right\}}=\exp^{\lambda }.$$

For instance, setting λ=0.7, the probability of maintaining edge status is almost twice the probability of not maintaining edge status between the subgroup and individual networks. These odds increase to about 20 and then 148 for λ=3 and λ=5, respectively.

Defining $p\left(M_{i}|y\right)=\prod_{r=1}^{n}p\left(M_{i}\left(r\right)|y\right)$, the MAP estimator of individual networks is then

$${\overset{ˆ}{M}}_{i}≔{arg max}_{M_{i}\in M_{i}}p\left(M_{i}|y\right).$$

The individual network structure for subject i can thus be found using the scores in Eq. (5) and MDM-IPA or MDM-DGM.

*MEMN: Subgroup Graphical Structures:* ${\overset{¯}{M}}_{1},\ldots ,{\overset{¯}{M}}_{G}$

The subgroup network is found through the posterior probability of ${\overset{¯}{M}}_{g}\left(r\right)$, as follows (see details in Appendix C).

$$p\left({\overset{¯}{M}}_{g}\left(r\right)|y\right)\propto \underset{i\in S_{g}}{\prod }\underset{M_{i}\left(r\right)\in M_{i}\left(r\right)}{\sum }\left[p\left(y_{i}|M_{i}\left(r\right)\right)\times p\left(M_{i}\left(r\right)|{\overset{¯}{M}}_{g}\left(r\right)\right)\right].$$

As $p\left({\overset{¯}{M}}_{g}|y\right)=\prod_{r=1}^{n}p\left({\overset{¯}{M}}_{g}\left(r\right)|y\right)$, the subgroup network structure is found using these scores above and MDM-IPA or MDM-DGM, so that

$${\overset{ˆ}{\overset{¯}{M}}}_{g}≔{arg max}_{{\overset{¯}{M}}_{g}\in {\overset{¯}{M}}_{g}}p\left({\overset{¯}{M}}_{g}|y\right).$$

*MEMN: Group Graphical Structure:* $\overset{¯}{M}$

The estimation of group network structure $\overset{¯}{M}$ using the individual networks $M_{1},\ldots ,M_{S}$ follows the same idea shown above for subgroup networks. Thus

(7) $$p\left(\overset{¯}{M}\left(r\right)|y\right)\propto \overset{S}{\underset{i=1}{\prod }}\underset{M_{i}\left(r\right)\in M_{i}\left(r\right)}{\sum }\left[p\left(y_{i}|M_{i}\left(r\right)\right)\times p\left(M_{i}\left(r\right)|\overset{¯}{M}\left(r\right)\right)\right],$$

and $p\left(\overset{¯}{M}|y\right)=\prod_{r=1}^{n}p\left(\overset{¯}{M}\left(r\right)|y\right)$. The MAP estimator is

$$\overset{ˆ}{\overset{¯}{M}}≔{arg max}_{\overset{¯}{M}\in \overset{¯}{M}}p\left(\overset{¯}{M}|y\right).$$

*Comparing the MEMN with the IEMN in Theory*

In IEMN, the individual networks are estimated independently, assuming that subjects have different networks, and so the information of one subject is not used in the estimation of another subject. In contrast, the group network is estimated considering that all subjects come from the same population and so they share the same graphical structure.

The higher the λ, the more similar the group network results are, comparing IEMN with MEMN. This is due to the fact that IEMN assumes that all subjects have the same graphical structure to estimate $\overset{¯}{M}$. Similarly, in MEMN, the higher the λ, the higher the score in which the distance between the individual and the group network is small, and so the more similar the individual graphs are to each other.

The smaller the λ, the more similar the individual results are ($M_{1},\ldots ,M_{S}$), when comparing IEMN with MEMN. When λ=0, $p\left(M_{i}\left(r\right)|{\overset{¯}{M}}_{g}\left(r\right)\right)$ is proportional to a constant, for i=1,…,S, and so $p\left(M_{i}\left(r\right)|y\right)$ is function of $c_{i}\left(r,M_{i}\left(r\right)\right)$ (see Eq. (5)). Therefore, the estimated individual graphical structures using IEMN are the same as those using the MEMN. In contrast, as λ increases, the scores are more penalised for individual networks that are more different from ${\overset{¯}{M}}_{g}$, therefore increasing the divergence between the IEMN and MEMN results.

Note that the comments above about group network $\overset{¯}{M}$ also apply to the subgroup networks ${\overset{¯}{M}}_{g}$. Some properties cited above will be demonstrated in the next section.

### 6.2 The application of multiple networks

We next use a rich fMRI study that has information from five different experimental conditions, called sessions: Session 1 is a (conventional) resting-state condition; session 2 is a motor condition in which individuals tapped something; session 3 is a visual condition in which individuals watched a movie; session 4 and session 5 are a combination between visual and motor condition, but the former is in a random way whilst in the latter individuals tap when they see certain random events in the movie. Data were acquired on 15 subjects, and each acquisition consists of 230 time points, sampled every 1.3 s, with 2 × 2 × 2 mm${}^{3}$ voxels. The FSL software^1^ was used for preprocessing, including head motion correction, automated artefact removal procedure Salimi-Khorshidi et al. (2014), Griffanti et al. (2014) and intersubject registration. We use 11 ROI’s defined on 5 *motor* brain regions and 6 *visual* regions. The motor nodes used are Cerebellum, Putamen, Supplementary Motor Area (SMA), Precentral Gyrus and Postcentral Gyrus (nodes numbered from 1 to 5, respectively) whilst the visual nodes used are Visual Cortex V1, V2, V3, V4, V5 and task negative (V1 + V2; nodes numbered from 6 to 11, respectively). The observed time series are computed as the average of BOLD fMRI data over the voxels of each of these defined brain areas (Costa et al., 2017).

In this section we discuss the main differences between IEMN and MEMN, highlighted in the previous section, considering now a real application. In addition, we explore some new methods for determining λ and discuss the impact of its values on the results of multi-subject analyses. We show that depending on the chosen value of λ, IEMN and MEMN can provide completely different results. It should be remembered that the space of λ parameter is (0,∞), *i.e.* it ranges from individuals believed to have different connectivity maps to hypotheses that they have the same one.

Some possibilities for defining this parameter are (i) through a scientific belief statement (Oates, 2013), *e.g.*, as shown in Section 6.1.2, λ=0.7 implies that the probability of maintaining edge status (absent/present) is almost twice the probability of not maintaining edge status between the group and the individual networks; (ii) maximising the LPL (or equivalently, maximising the scores $c_{i}\left(r,M_{i}\left(r\right)\right)$ or $c(r,\overset{¯}{M}\left(r\right)$); (iii) maximising the posterior probability of individual networks, $p\left(M_{i}|y\right)$, or group networks, $p\left(\overset{¯}{M}|y\right)$; (iv) by cross-validation. We show here that different ways of estimating λ can lead to very different values of this parameter, and so divergent analyses result.

In this section we also compare MDM-IPA and MDM-DGM. Here we show that the graphs estimated by MDM-DGM are usually denser than DAGs from MDM-IPA, to accommodate for the possible cycles in the communication among brain regions. We also show that the methods described here can be used to compare data from different experimental conditions.

For the purposes described above, we are using an external validation study, in which the estimated individual networks were compared to predictive networks, *i.e.* networks estimated using the data from other S−1 subjects. For simplicity, we are considering two levels: the individual and the group network. However, this analysis could of course also be applied to subgroup networks as well.

First the individual networks were estimated considering IEMN and MEMN, with λ=0.1,0.7,10,100 and 1000, using MDM-IPA and MDM-DGM. Then the predicted individual network for subject i was estimated considering the group analysis for all subjects, except subject i, using the same methods as before.

**Fig. 8:**
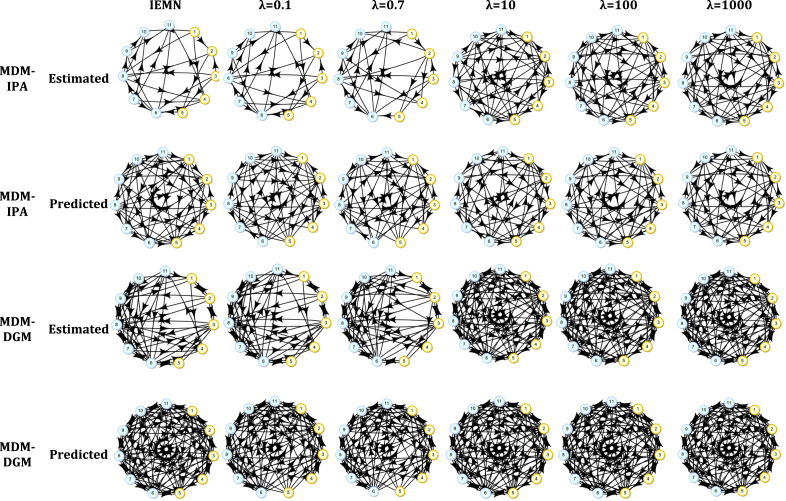
The estimated and predicted networks, using the MDM-IPA and the MDM-DGM, for subject 1 and resting-state condition (Session 1), considering IEMN (the first column) and MEMN (from the second column) with λ=0.1,0.7,10,100 and 1000. The motor nodes used are Cerebellum, Putamen, Supplementary Motor Area (SMA), Precentral Gyrus and Postcentral Gyrus (yellow nodes numbered from 1 to 5, respectively) whilst the visual nodes used are Visual Cortex V1, V2, V3, V4, V5 and task negative (v1 + v2; blue nodes numbered from 6 to 11, respectively).

Fig. 8 shows the estimated and predicted graphical structures for subject 1 in the resting-state condition, considering all the methods described above. As expected, the estimated individual network found using IEMN was similar to the one using MEMN with small λ (see the first and second column and the first and third row). In contrast, as the predicted results were found using the methods of group network and as discussed above, the predicted network of IEMN was similar to the MEMN one with large λ (see the first and last column and the second and last row).

The parameter λ can be defined maximising the individual $c_{i}\left(M_{i}\right)$ and the group $c\left(\overset{¯}{M}\right)$ scores. They were evaluated as a function of the posterior probability of individual and group networks, by Eq. (5) and Eq. (7) , respectively, *i.e.*

$$c_{i}\left(M_{i}\right)≔\log p\left(M_{i}|y\right)=\overset{n}{\underset{r=1}{\sum }}\log p\left(M_{i}\left(r\right)|y\right),$$

$$c\left(\overset{¯}{M}\right)≔\log p\left(\overset{¯}{M}|y\right)=\overset{n}{\underset{r=1}{\sum }}\log p\left(\overset{¯}{M}\left(r\right)|y\right).$$

The value of λ that maximised these scores was 0.7 for both individual and group networks, and also for MDM-IPA and MDM-DGM (see Table 2).

 Now analysing the number of edges, the results also clearly depend on the individual and the group networks. We can see in Fig. 8 that the higher the λ, the denser the estimated individual networks were (the first and third row) whilst the group networks got sparser with the growth of λ (the second and last row).

**Table 2:** The average of scores for individual networks, $c_{i}\left(M_{i}\right)$, and group networks, $c\left(\overset{¯}{M}\right)$, over subjects and sessions, considering the learning network algorithms: MDM-IPA and MDM-DGM, and different values of λ.

λ	Individual		Group	
	IPA	DGM	IPA	DGM
0.1	20 336.50	20 851.97	19 414.24	19 426.27
0.7	20 382.76	20 904.73	19 423.86	19 507.02
10	19 929.91	20 731.38	18 119.40	19 348.62
100	18 844.87	20 729.59	14 671.61	19 347.24
1000	14 349.80	20 729.59	13 499.95	19 347.24

**Fig. 9:**
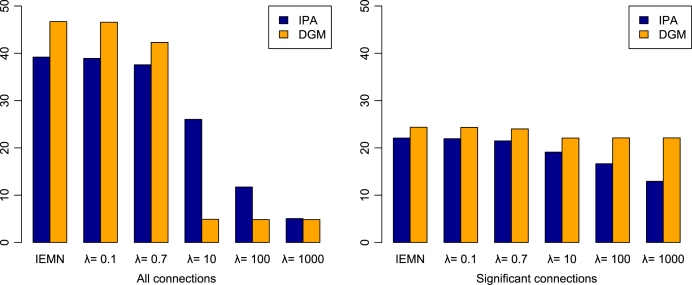
The average of the structural Hamming distance comparing two individual networks over all pairwise subjects and sessions, considering IEMN and the MEMN (λ=0.1,0.7,10,100,1000), using the MDM-IPA (blue bars) and the MDM-DGM (orange bars), for the all edges (left) and the only significant edges (right) of estimated networks. (For interpretation of the references to colour in this figure legend, the reader is referred to the web version of this article.)

We also study which method provides graphical structures which are more similar over subjects. Fig. 9 shows the average of the SHD between two estimated individual networks, over all pairwise subjects and all sessions, considering IEMN and MEMN (λ=0.1,0.7,10,100,1000), using the MDM-IPA (blue bars) and the MDM-DGM (orange bars). Considering the complete individual graphical structure (Fig. 9, left), IEMN and the small values of λ for MEMN provided different results across subjects. This result is expected as long as the large λ implies to a similar structure between the individual networks ($M_{i}$’s) and the group network ($\overset{¯}{M}$), and then, among the individual graphs. In this way, the MEMN with large λ is suitable for a homogeneous group. This conclusion is confirmed considering the estimated individual graphs, with only the significant connections (right figure), although the difference between the methods is faint.

**Fig. 10:**
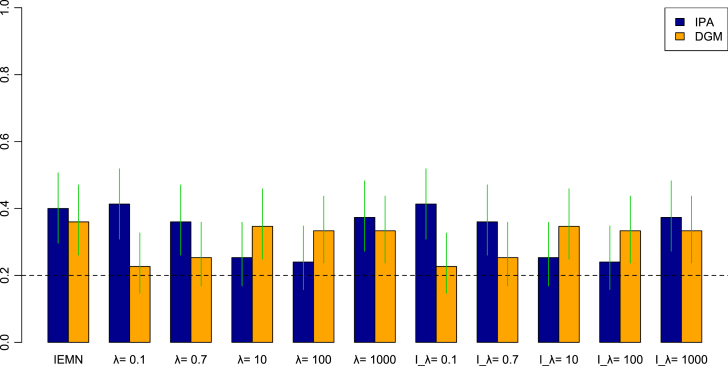
The average of the percentage of predicting correctly the network session, over all sessions, comparing the estimated to the predicted networks using the same method, *i.e.* IEMN and MEMN with λ=0.1,0.7,10,100,1000, and comparing the estimated networks using IEMN to the predicted networks using MEMN for l_λ=0.1,0.7,10,100,1000, using the MDM-IPA (blue bars) and the MDM-DGM (orange bars). The dashed horizontal line means the chance of predicting correctly the network session randomly. The green lines represent the 95% HPD intervals, considering a non-informative prior distribution. (For interpretation of the references to colour in this figure legend, the reader is referred to the web version of this article.)

The estimated and the predicted networks were compared using the logBF and the SHD, for the complete graph and considering only significant edges. We then computed the percentage of subjects in which the distance between the estimated and the predicted is the smallest when both networks belong to the same session. Fig. 10 provides the average of this percentage of predicting correctly the network session, over all sessions, comparing the estimated to the predicted networks using the same method, *i.e.* IEMN and MEMN with λ=0.1,0.7,10,100,1000, and comparing the estimated networks using the IEMN (*i.e.* individual graphs estimated independently) to the predicted networks using the MEMN for λ=0.1,0.7,10,100,1000 (we called this l_λ). The chance of predicting the correct network session randomly is 1∕5 sessions (dashed horizontal line in this figure). The green lines represent the 95% HPD intervals, when employing a non-informative prior distribution. In general, IEMN and MEMN with λ=1000 provided one of the best results. For MDM-IPA, MEMN with λ=0.1 had the highest percentage of predicting the network session (around 40%) correctly. Overall, MDM-IPA predicted the network session more correctly than MDM-DGM.

To compare sessions, we identified which sessions were better at predicting another particular session. For instance, Table 3 shows sessions (columns 3 and 5) that have the highest percentage of predicting the network of session cited in column 1, considering the method MEMN l_λ=1000. Considering all methods and different values of λ, in general,

• Session 1, resting-state condition, better predicted Session 2, motor condition;
• Session 3, visual condition, and Session 4, visual and motor (random) condition, better predicted each other;
• Session 3 also better predicted Session 5, visual and motor condition;
• There was not a predominant session that predicted Session 1, resting-state.

Note that these results are consistent with the conclusion provided in Costa et al. (2017), where Session 1, resting-state, was responsible for the greatest difference among sessions considering the connectivity maps, and Session 3, visual condition, is closer to Session 4, visual and motor conditions, than to other sessions.

**Table 3:** The percentage of subjects who have a particular session chosen by the smallest value of the logBF comparing estimated network, using IEMN, to the predicted network, using MEMN with λ=1000. Columns 2 and 4 show this percentage regarding the same session as in column 1, for MDM-IPA and MDM-DGM, respectively. Columns 3 and 5 show the session (and the correspondent percentage) that better predicts the session in column 1, whereas all other sessions, for MDM-IPA and MDM-DGM, respectively.

Session	MDM-IPA		MDM-DGM	
	% right session	Predictor session (%)	% right session	Predictor session (%)
1-RS	40%	5 (33%)	47%	3 (27%) and 4 (27%)
2-Motor	47%	1 (33%)	33%	1 (27%)
3-Visual	33%	4 (40%)	40%	4 (33%)
4-Visual + Motor (random)	40%	3 (33%)	33%	1 (40%)
5-Visual + Motor	27%	3 (40%)	13%	4 (47%)

## 7 Conclusions

Many experimental designs in neuroscience involve data collected on multiple subjects. They may differ with respect to neural connectivity, such that corresponding graphs $M_{i}$ may be subject-specific Sugihara et al. (2006), Li et al. (2008). The elements of neural architecture are assumed to be largely conserved among subjects. Therefore it is natural to leverage this similarity in order to improve statistical efficiency by addressing both the robustness of inferred graphical structure and reducing small sample bias (Mechelli et al., 2002). The statistical challenge of estimating multiple related graphical models has recently received much attention, *e.g.* the VTS, the CS and the IS approaches Mechelli et al. (2002), Zheng and Rajapakse (2006), Rajapakse and Zhou (2007), Li et al. (2008), Ramsey et al. (2010). Table 4 clarifies the main difference in the assumptions of all the methods discussed in this work.

Here VTS was applied to evaluate the average of time series variables over subjects. Although this method performed poorly in the synthetic study, it was consistent with the other methods for the real fMRI data. The CS approach provided dense graphs, in both synthetic and real studies. However, in the sub-groups of simulated data, where most of the subjects share the same graphical structure, the process of summing scores had excellent performance. The IS approach provided sparser graphs than the other methods, and its result appeared to reflect what happened to most subjects.

Studies suggest that it might be possible to increase statistical efficiency, often considerably, by formulating an appropriate joint model that couples multiple graphs. However, these methods use an exchangeability assumption, treating the entire group as homogeneous. Therefore these approaches are bound to provide inconsistent results for a heterogeneous group, as shown above. In our study, we saw that only the GS approach can recognise the heterogeneity that existed in the group.

We also developed the IEMN and MEMN methods based on the GS approach. We showed that these two approaches provided similar results when the hyperparameter λ is small for individual networks and large for group networks. Moreover, the higher the λ, the denser the individual networks are, but the sparser the group networks are. We also discussed some procedures that can be applied in order to estimate λ. The results found here suggest that the estimation of λ depends on the aim of the study, *e.g.*, if one wishes to predict the connectivity for a new subject, then cross-validation can be used, but if the focus is on estimating individual networks, then λ can be chosen maximising the posterior distribution, $p\left(M_{i}|y\right)$. In general, the appropriate choice of λ is also related to how homogeneous the group is, and so how much of the information of other datasets should also be included in the analysis of one dataset.

As the aim of this work is to discuss methods that deal with multiple networks and in principal these approaches can be used with other graphical models, we do not explore methods applied to fMRI data. However, a reader interested in this subject can see a comparison between the MDM and other methods usually applied to neuroscience data (*e.g.* Granger causality, Linear Dynamic System and Patel’s τ) in Costa et al. (2015) and a recent discussion about state-space methods used to study dynamic networks with application into fMRI data in Solo (2016).

**Table 4:** The main assumptions for all methods described in this work. Note that IEMN and MEMN are based on the GS approach.

Assumptions	VTS	CS	IS	IEMN	MEMN
The degree of similarity between subjects is fixed	√	√	√	√	
Homogeneity	√	√	√		
The same graphical structure	√	√			
The same connectivity strengths	√				

## Appendix A Short description and some references for all methods used in this work

**The Multiregression Dynamic Model (MDM):**: It is a class of multivariate time series models which embeds putative causal hypotheses among its variables over time Queen and Smith (1993), Queen and Albers (2009), Costa et al. (2015);
**The Multiregression Dynamic Model-Integer Programming Algorithm (MDM-IPA):**: It is an efficient search-and-score method that provides a search over the large class of networks based on an integer programming algorithm Bartlett and Cussen (2013), Costa et al. (2015);
**The Multiregression Dynamic Model-Directed Graph Model (MDM-DGM):**: It is also a learning network algorithm but searchesfor graphical structure without the constraints of DAG (Costa et al., 2017);
**The Virtual-typical-subject (VTS) Approach:**: It ignores inter-subject variability, assuming that the information from different datasets come from the same subject Zheng and Rajapakse (2006), Rajapakse and Zhou (2007), Li et al. (2008);
**The Common-structure (CS) Approach:**: It considers the same network structure but allows the parameters to differ between subjects Ramsey et al. (2010), Li et al. (2008);
**The Individual-structure (IS) Approach:**: It drives the learning network process individually in each dataset so that results are pooled into a single network Mechelli et al. (2002), Li et al. (2008), Oates (2013);
**The Group-structure (GS) Approach:**: It studies group homogeneity through cluster analysis, considering a particular measure of similarity between subjects Kherif et al. (2004), Gates (2012);
**The Individual Estimation of Multiple Networks (IEMN) Approach:**: It is basically the GS approach, *i.e.* the individual networks are estimated independently whilst the subgroup networks made up of homogeneous subjects are estimated as a function of the same graphical structure for all individuals Kherif et al. (2004), Gates (2012);
**The Marginal Estimation of Multiple Networks (MEMN) Approach:**: It searches both individual and subgroup networks considering a distance between homogeneoussubjects Oates (2013), Oates et al. (2014a), Oates et al. (2014b).

## Appendix B Individual graphical structures for the MEMN

(8) p(Mi(r)|y)=∑M¯g(r)∈M¯g(r)∑Mk(r)∈Mk(r)k∈Sg∖{i}p(M¯g(r),M1∗(r),…,MSg∗(r)|y)∝∑M¯g(r)∈M¯g(r)∑Mk(r)∈Mk(r)k∈Sg∖{i}[p(y|M1∗(r),…,MSg∗(r))×p(M1∗(r),…,MSg∗(r)|M¯g(r))×p(M¯g(r))]∝∑M¯g(r)∈M¯g(r)∑Mk(r)∈Mk(r)k∈Sg∖{i}∏l∈Sg[p(ylT|Ml(r))×p(Ml(r)|M¯g(r))]=∑M¯g(r)∈M¯g(r)[p(yiT|Mi(r))×p(Mi(r)|M¯g(r))×∏k∈Sg∖{i}∑Mk(r)∈Mk(r)p(ykT|Mk(r))×p(Mk(r)|M¯g(r))],

where $p\left({\overset{¯}{M}}_{g}\left(r\right)\right)$ here is proportional to a constant because we are assuming that a priori all subgroup network structures are equally probable.

## Appendix C Subgroup graphical structures for the MEMN

p(M¯g(r)|y)=∑Mi(r)∈Mi(r)i∈Sgp(M¯g(r),M1∗(r),…,MSg∗(r)|y)∝∑Mi(r)∈Mi(r)i∈Sg[p(y|M1∗(r),…,MSg∗(r))×p(M1∗(r),…,MSg∗(r)|M¯g(r))×p(M¯g(r))]∝∏i∈Sg∑Mi(r)∈Mi(r)[p(yi|Mi(r))×p(Mi(r)|M¯g(r))].

Again $p\left({\overset{¯}{M}}_{g}\left(r\right)\right)$ is considered as proportional to a constant.

## Footnotes

1 http://fsl.fmrib.ox.ac.uk.
